# Local Barriers and Solutions to Improve Care-Seeking for Childhood Pneumonia, Diarrhoea and Malaria in Kenya, Nigeria and Niger: A Qualitative Study

**DOI:** 10.1371/journal.pone.0100038

**Published:** 2014-06-27

**Authors:** K. Juliet A. Bedford, Alyssa B. Sharkey

**Affiliations:** 1 Anthrologica, Oxford, Oxfordshire, United Kingdom; 2 School of Anthropology, University of Oxford, Oxford, Oxfordshire, United Kingdom; 3 Knowledge Management and Implementation Research Unit, Health Section, Program Division United Nations Children's Fund (UNICEF), New York, New York, United States of America; The George Washington University Medical Center, United States of America

## Abstract

We present qualitative research findings on care-seeking and treatment uptake for pneumonia, diarrhoea and malaria among children under 5 in Kenya, Nigeria and Niger. The study aimed to determine the barriers caregivers face in accessing treatment for these conditions; to identify local solutions that facilitate more timely access to treatment; and to present these findings as a platform from which to develop context-specific strategies to improve care-seeking for childhood illness. Kenya, Nigeria and Niger are three high burden countries with low rates of related treatment coverage, particularly in underserved areas. Data were collected in Homa Bay County in Nyanza Province, Kenya; in Kebbi and Cross River States, Nigeria; and in the Maradi and Tillabéri regions of Niger. Primary caregivers of children under 5 who did not regularly engage with health services or present their child at a health facility during illness episodes were purposively selected for interview. Data underwent rigorous thematic analysis. We organise the identified barriers and related solutions by theme: financial barriers; distance/location of health facilities; socio-cultural barriers and gender dynamics; knowledge and information barriers; and health facility deterrents. The relative importance of each differed by locality. Participant suggested solutions ranged from community-level actions to facility-level and more policy-oriented actions, plus actions to change underlying problems such as social perceptions and practices and gender dynamics. We discuss the feasibility and implications of these suggested solutions. Given the high burden of childhood morbidity and mortality due to pneumonia, diarrhoea and malaria in Kenya, Nigeria and Niger, this study provides important insights relating to demand-side barriers and locally proposed solutions. Significant advancements are possible when communities participate in both problem identification and resolution, and are engaged as important partners in improving child health and survival.

## Introduction

Pneumonia, diarrhoea and malaria remain the three largest killers of children under 5 and together account for one third of child deaths worldwide [1]. Simple, inexpensive treatments are available for each of these conditions, yet too few children receive appropriate and timely care, particularly in high burden countries and in the most deprived settings due to a range of interrelated factors. These include insufficient supply of high quality health commodities and trained health workers, poor geographic access to services, poor quality of care, as well as user-related financial and non-financial barriers [2]. As has been well documented, in sub-Saharan Africa these user-related barriers are locally specific, and can include cultural beliefs; perceptions of illness; illness severity and efficacy of treatment; social norms around household decision-making and gender; location; household income and direct and in-direct costs of treatment; and perceptions of and previous experiences with health services [3], [4], [5], [6]. In many countries, these supply- and demand-related barriers have had a significant impact on progress towards the achievement of Millennium Development Goal (MDG) 4 [7], [8].

We present the results of qualitative research to identify demand-related barriers and solutions to care-seeking and treatment uptake for pneumonia, diarrhoea and malaria among children under 5 years of age in low-resource settings in Kenya, Nigeria and Niger. In these countries, UNICEF is supporting its government partners with focused efforts to improve treatment coverage through a range of policy and programmatic strategies, including the expansion of integrated community case management for pneumonia, diarrhoea and malaria. The research was designed to support government-stated policy priorities. For example, the Kenya Health Policy 2012–2017 includes a central goal to attain ‘the highest possible health standards in a manner responsive to the population needs’ and includes action to ‘improve evidence-based decision-making and resource allocation’ and ‘review and realign community based services around expectations’ as key recommendations [9]. In Nigeria, ‘community participation and ownership’ is a priority area of the National Strategic Health Development Plan 2011–2015 and ‘increasing access to’ and ‘demand for health services’ are key objectives [10]. In Niger, the *Plan de Developpement Sanitaire* 2011–2015 includes enhancing community participation in the development and monitoring of health programmes and strategies as a key objective [11].

The research was based on thematic analysis using grounded theory. The objectives were to a) determine the barriers and challenges families face in accessing treatment for childhood pneumonia, diarrhoea and malaria; b) identify local solutions that promote and facilitate more timely access to appropriate treatment for these conditions; and c) present these findings as a platform from which to develop context-specific strategies to increase demand and improve care-seeking for childhood illness. Building on knowledge gained from previous studies on barriers to care-seeking in Africa, this research provides new information regarding specific barriers faced by care-givers in these settings and, importantly, elicits relevant and locally acceptable programmatic solutions to the barriers through the active engagement of community members. Although not in itself a main objective of the research, the use of this participatory approach also informed UNICEF's related programmatic efforts to improve coverage of key health interventions in under-performing districts across Africa.

## Methods

### Setting

Kenya, Nigeria and Niger, were selected as the sites of this research because of their high burden of childhood pneumonia, diarrhoea and malaria, low rates of related treatment coverage (table 1) and because they were identified by UNICEF as key countries to target concerted efforts to reduce childhood mortality. UNICEF is working closely with the governments of the three countries. Each has identified the improvement of treatment for these childhood illnesses as a national priority and has designated specific, underserved focal regions for intensive intervention scale up. The research was conducted in several of these regions in order to provide a rounded evidence-base.

**Table 1:** The burden of diarrhoea, pneumonia and malaria and related indicators, Kenya, Niger and Nigeria (various years).

	Kenya	Niger	Nigeria
Infant mortality rate (per 1000 live births, 2012)^a^	49	63	78
Under 5 mortality rate (per 1000 live births, 2012)^a^	73	114	124
Percent of all deaths among children under 5 due to (2010):^b^			
Diarrhoea	15	11	11
Pneumonia	22	17	17
Malaria	15	20	20
One-year-old children immunised against (percent, 2010):^c^			
Measles	86	71	71
Hib	83	70	–
DTP3	83	70	69
Infants under age 6 months who are exclusively breastfed (percent, 2006–2010)^c^	32	27	13
Children under age 5 who are (percent, 2006–2010):^c^			
Underweight (moderate and severe)	16	40	23
Stunting (moderate and severe)	35	47	41
Population using improved drinking water sources (percent, 2010):^d^			
Total	59	49	58
Urban	82	100	74
Rural	52	39	43
Population using improved sanitation facilities (percent, 2010):^d^			
Total	32	9	31
Urban	32	34	35
Rural	32	4	27
Ratio of urban to rural	1.0	8.5	1.3
Population using solid fuels as the main cooking fuel (percent, 2010)^c^	80	>95	74
Antibiotic treatment for suspected pneumonia among children under 5 (percent, 2007–2012)^d^	50	–	23
Antimalarial treatment among febrile children under 5 (percent, 2007–2012)^d^	23	–	49
Treatment with oral rehydration therapy (ORT) and continued feeding among children under 5 (percent, 2006–2009)^c^	43	34	25
Treatment with ORT and continued feeding among children under 5 by wealth quintile (percent, 2006–2009):^c^			
Richest 20%	41	46	41
Poorest 20%	49	31	17
Treatment with ORT and continued feeding among children under 5 by residence (percent, 2006–2009):^c^			
Urban	44	47	34
Rural	42	32	22

**Footnote for** **table 1** **:**

a ) UN Inter-agency Group for Child Mortality Estimation (2013) Levels & trends in child mortality.

Report 2013. New York, UNICEF. 30 p.

b ) Child Health Epidemiology Reference Group.

Child causes of death annual estimates by country, 2000–2010. Unpublished estimates available at http://cherg.org/datasets.html. Accessed 31 May 2013.

c ) UNICEF (2012) Pneumonia and diarrhoea: tackling the deadliest diseases for the world's poorest children.

UNICEF: New York. 77 p.

d ) UNICEF (2013) State of the world's children 2013.

Children with disabilities. UNICEF: New York. 164 p.

**Figure 1:**
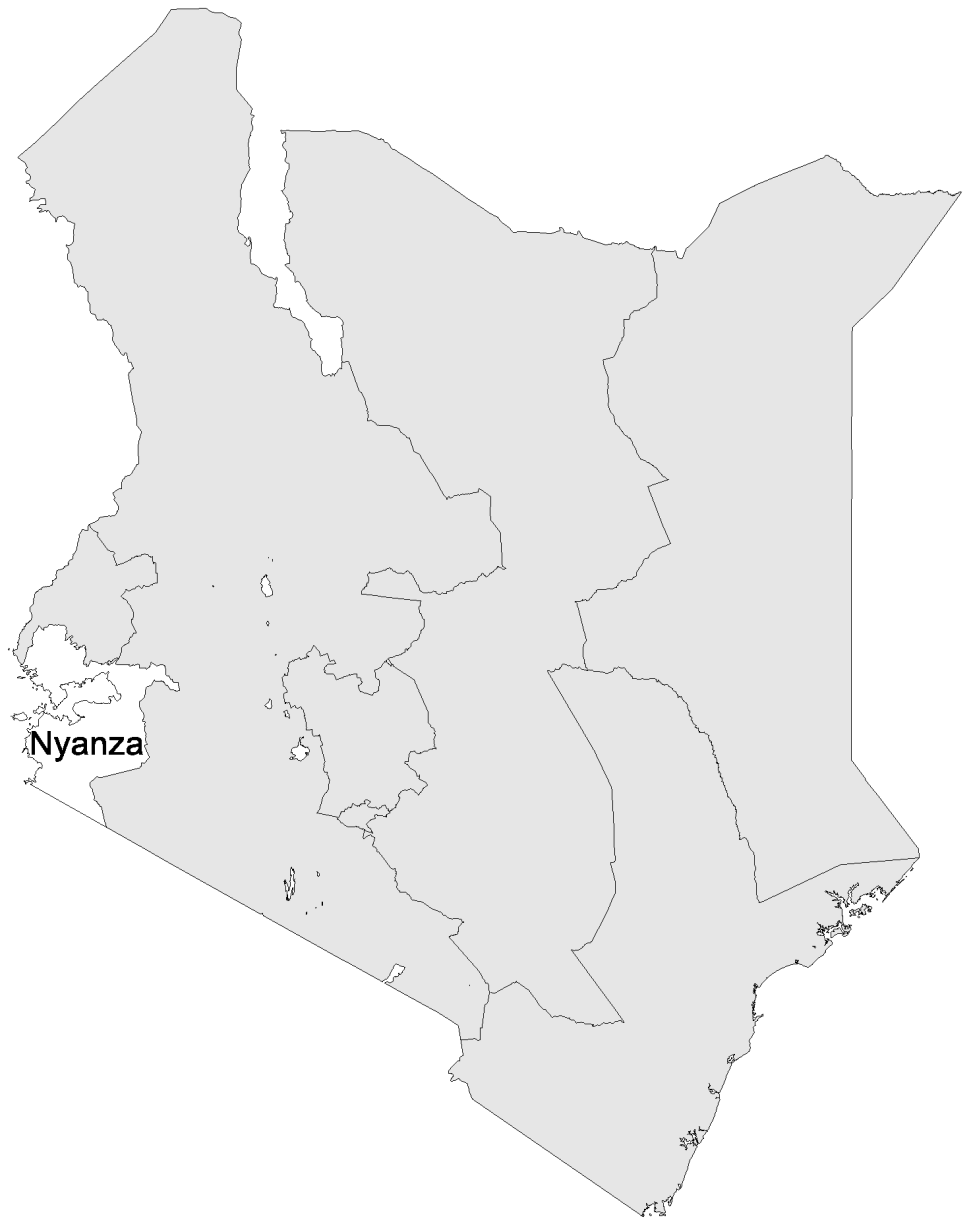
Map of Kenya fieldsite.

In Kenya, the study focused on Homa Bay County in Nyanza Province (figure 1). In 2009, the Province, which borders with Lake Victoria in the West of Kenya, had the highest under 5 mortality rate in the country at 149 per 1,000 live births, representing 33,826 deaths annually, or 33% of all child deaths in Kenya [12]. Homa Bay County is primarily a rural area with limited geographic access to health facilities. Data collection was conducted over twelve days in May-June 2012 in three specific fieldsites agreed in collaboration with the Homa Bay District Health Management Team: Ndiru (Homa Bay District); Marindi (Ndhiwa District); and Lambwe (Mbita District). Each of these sites included remote communities that were reachable only by foot.

No previously published studies on barriers to care-seeking for childhood illnesses in Homa Bay county were identified, yet studies from neighbouring rural districts in western Kenya suggest that key barriers include a lack of knowledge about available services and a preference for traditional healers [13]; distance to facilities, associated costs and the perception of poor services [14]; and low socioeconomic status and maternal perceptions of illness severity [15].

**Figure 2:**
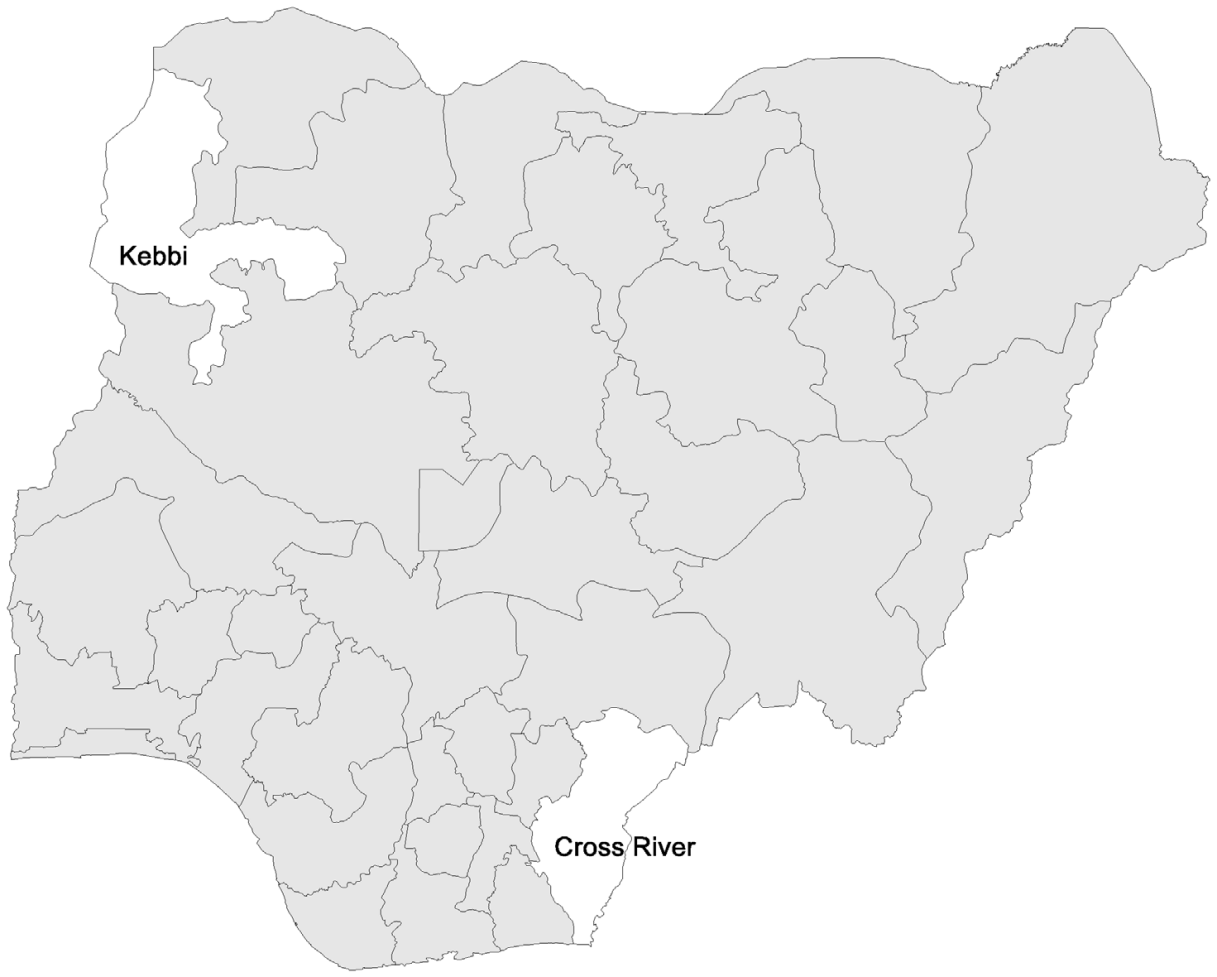
Map of Nigeria fieldsites.

In Nigeria, the study was located in two diverse states: Kebbi and Cross River (figure 2). In 2008, the under 5 mortality rate was 217 per 1000 live births in the North-West Zone where Kebbi is located, and 153 per 1000 in the South-East Zone where Cross River is located [16]. The two states are socio-culturally and geo-politically very different, and the findings reflect important variations between the communities. Kebbi borders Niger and Benin and is part of the dry and arid Sahel region. It is populated mainly by Hausa-speaking peoples and is Islamic. The practice of *kulle* (literally meaning ‘shut-in’), where women are restricted to the home, is widespread. Cross River borders Cameroon. With its warm climate and tropical rainforest, it is often billed as Nigeria's primary tourist destination and remains an important trade route. It is predominantly Christian, and home to a number of different ethnic groups including the Efik, Ejagham and Bekwarra. Data collection in both states was conducted over ten days in July 2012. Specific fieldsites were agreed in collaboration with the Ministry of Health in each state. In Kebbi, four Local Government Areas (LGAs) were visited: Koko (Hilin Harisu and Wurya settlements), Augie (Tiggi village), Gwandu (Tamawah and Kuraigishiri villages) and Birnin Kebbi (Takalau and Kofar Kola settlements). In Cross River, four LGAs were also visited: Obubra (Lyamoyong village), Abi (Ebom village), Yakurr (Mkpani village) and Akampa (Oban village). Koko and Bernin Kebbi are urban centres, the others are rural towns. No fieldsite was extremely remote and all were accessible by road.

Although a large body of research has been conducted in Nigeria that relates to care-seeking for childhood illnesses more broadly, only one relevant published study was identified for Cross River State [17] and none were identified for Kebbi State. The Cross River study identified two key influencers: socio-cultural considerations dictating that a mother must consult in-laws or her husband before seeking care outside the home; and living in an extended family network (which increases the likelihood of administering home-based treatments such as herbs or a plain water enema). Studies from other areas within Nigeria have found that financial barriers, distance from home to facility, failure to recognise danger signs (such as fever), and preference for traditional remedies also play a role [18], [19], [20], [21], [22].

**Figure 3:**
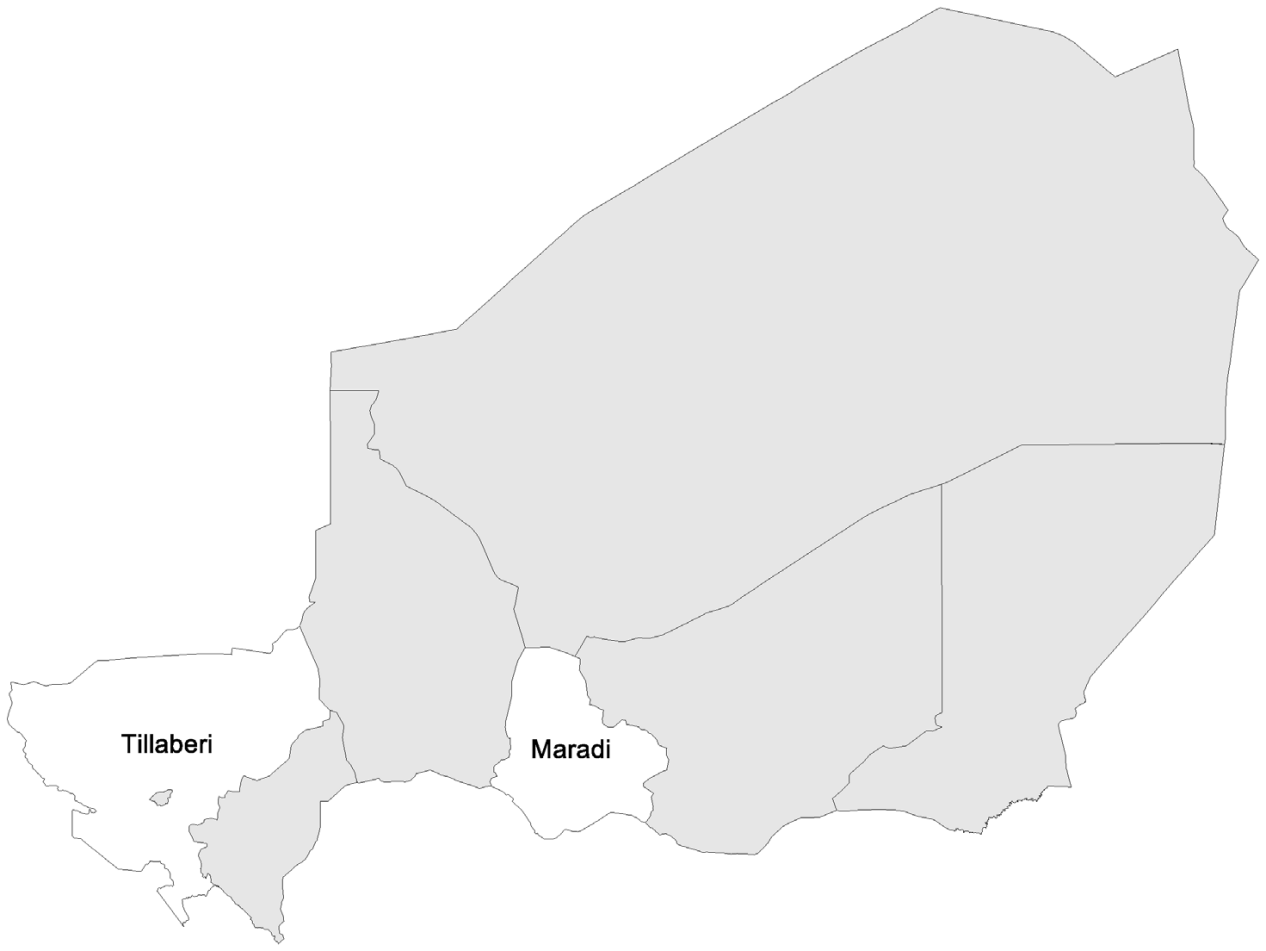
Map of Niger fieldsites.

In Niger, two study sites were selected to provide information across different health system performance contexts: Madarounfa District in the Maradi Region, and Kollo District in the Tillabéri Region (figure 3). Maradi borders with Nigeria and had an under 5 mortality rate of 231 in 2006, the most recent year for which local data were available [23]. The Tillabéri region borders with Mali and Burkina Faso and had an under 5 mortality rate of 193 in 2006, the most recent year for which local data were available [24]. Data collection in both districts was conducted over ten days in August 2012 and specific fieldsites were agreed in collaboration with the District Health Teams of Madarounfa and Kollo. In Madarounfa, five villages were visited: Soumarana, Bargaja, Dan Mazadou, Radi and Samia. In Kollo, four villages were visited: Goubé, Bokotchilli, Bartchawal and Lelehi Mamane Gnalli. No fieldsite was extremely remote and all villages were accessible by road.

There are few published studies that relate to care-seeking for childhood illness in Niger. One study from Niger's Maradi region explored care-seeking behaviour for diarrhoea [24]. Caregivers in this study readily recognised diarrhoea symptoms and formal care-seeking at health facilities was very high. Among those who did not seek care, most cited spontaneous recovery or self-medication obtained from drug vendors as the key reasons. Further, around 10% of caregivers cited financial problems as the main constraint. Another study that investigated antibiotic regimen adherence for pneumonia in the Dosso region [25] examined a range of determinants including parental knowledge of respiratory infections, medications, traditional remedies and health-seeking behaviour; cultural beliefs about wellness and illness; traditional dissemination of information; and the appropriate way to deliver messages to caregivers. The findings revealed that parents had difficulty remembering instructions they were given about how to administer antibiotics and they often discontinued treatment prematurely. The study also revealed that antibiotics were available more cheaply at markets than health facilities, so caregivers often purchased medication from the informal sector.

Achieving the MDG target for under 5 mortality remains a challenge for each of these three countries [26]. Niger has made the most significant progress: its under 5 mortality rate has fallen 43% in a decade, from 226 per 1,000 in 1998 to 128 per 1,000 in 2009 [27] and it is amongst 23 of the 74 Countdown countries on track to achieve its MDG 4 [28]. Unpublished data from the *Système National d'Information Sanitaire* (SNIS, Niger Health Information System) indicates an increase in the utilisation of health services during the same period for malaria, diarrhoea and pneumonia. Despite this, challenges and barriers to effective care-seeking and treatment uptake remain in Niger, as in Kenya and Nigeria.

### Research team

The research was led by the primary author (JB), supported by national research assistants in each country. JB has a PhD in anthropology and is the Director of Anthrologica, a research-based organisation specialising in applied anthropology in global health. National research assistants participated in interviews and focus group discussions (FGDs) as translators and were also responsible for transcribing the audio recordings of interviews and discussions held. Logistical support was provided by UNICEF country offices and the head office in New York. Alyssa Sharkey (AS) contributed to the conception and design of the study, as well as the cross-country results and implications. AS has a PhD in public health with specialisations in child survival and qualitative methods. Secondary analysis was undertaken at the conclusion of the research by an Anthrologica research associate.

### Participants and recruitment

156 participants were included in the research across the three countries: 65 in-depth interviews were conducted with primary carers of children under five; five FGDs with fathers of children under five; and four FGDs with health professionals (table 2).

**Table 2:** Overview of participants per data collection method in each study site.

	Kenya	Nigeria		Niger	
	Homa Bay	Cross River	Kebbi	Maradi	Tillabéri
Interviews with primary care givers	20	11	11	12	11
FGD with fathers of children under 5	1	1	1	1	1
FGD with health professionals	1	1	1	1	-
Total participants	39	35	31	30	21

In Kenya, the study collaborated with four community health units. The scope and objective of the research was outlined to the clinical officer, Community Health Extension Worker (CHEW) or member of the Community Unit Committee (CUC) attached to the health facility in each health unit. They appointed Community Health Workers (CHW) to liaise with the research team and make prior contact with participants. In Kebbi, Nigeria, the team collaborated with UNICEF communication consultants (employed as part of the polio eradication programme) and health educators. They arranged for Volunteer Community Mobilisers (VCMs) to introduce the research team to the community, and a representative from the State Ministry of Health accompanied the team to a selection of sites. In Cross River, Nigeria, the State Ministry of Health introduced the team to different local facilitators: a Principal Community Health Extension Worker, Assistant Chief Community Health Officer and a Cold Chain Officer. These personnel liaised with the communities and facilitated the team's introduction to key participants. In Niger, the District Health Teams contacted health personnel at health posts and/or the chief of each selected village in preparation for the team's visit. Various community members then facilitated the team's introduction to the community including CHWs, traditional birth attendants, members of the health post committee, and family members of the village chief.

For the in-depth interviews, local community health workers were asked to identify primary carers of children under five years old, who were purposively selected for interview if they reported to the research team that they did not regularly engage with health services or present their child at a health facility during illness episodes. Only one potential participant declined to be interviewed by the research team. Fathers included in the focus group discussions were selected by the local CHWs or village leader. The participants of three of the four FGDs with health professionals were the health workers with whom the study had collaborated. For logistical reasons, the FGD in Cross River, Nigeria, was with CHWs from an LGA not otherwise visited by the research team. These participants were selected and invited to attend the FGD by the Monitoring and Evaluation Disease Surveillance and Notification Officer of Akpabuyo LGA. In Niger, a FGD with health professionals was not facilitated in Tillabéri because the research team had collaborated with community members rather than health workers in that district.

### Data collection and security

Based upon a previous literature review by Colvin [5] and unpublished UNICEF data, JB devised a series of methodological tools including a topic guide that highlighted key issues and was the basis for the design of the semi-structured interview and FGD frameworks. These included a broad spectrum of research questions and probes (see Information S1 and Information S2). Specific questions and probes were reviewed and refined during the research period in light of themes arising. Although the direction of each interview was determined by the interviewee and largely focused on issues they self-prioritised (rather than on what the research team may have presupposed to be important), the key topics were addressed in each interview and therefore allowed generalisation of themes across participants.

All interviews were conducted by JB in English, with the research assistant translating sequentially between the appropriate local languages (in Kenya, Kswahili; in Nigeria, Hausa in Kebbi and Pidgin English in Cross River; in Niger, Hausa in Madarounfa, and Djerma in Kollo). Each interview lasted between one and two hours and audio recordings were made using a digital voice recorder. The focus groups were conducted in English and the local language, again with JB facilitating the discussion and the research assistant translating. Audio recordings were also made of the group discussions. Both JB and the research assistants made extensive notes during each interview and FGD.

To support data security, backup copies of transcriptions and audio recordings were made. Once all the files were finalised and saved on JB's computer (which was password protected), the research assistants deleted all files from their computers.

### Data analysis

At the conclusion of each day of data collection, the research team compiled and transcribed their interview and FGD notes. The audio recordings were transcribed in full with sections of narrative translated and back translated for quality assurance. Preliminary analysis was conducted in-country throughout the research process. Using an inductive approach, initial findings were discussed and at the conclusion of fieldwork in each country a roundtable debrief session was held between the research team and UNICEF country office technical specialists in health, social protection, and communication for development.

JB was responsible for the complete thematic analysis of the interviews using grounded theory [29], [30], [31]. Dominant themes were identified through the systematic sorting of data, labelling ideas and phenomena as they appeared and reappeared. Coding and analysis was done by hand. The emerging trends were analysed according to the research objectives using the critical-interpretive approach of medical anthropology [32], [33], [34]. At the conclusion of the research in all three countries, a second researcher from Anthrologica undertook analysis of a sub-set of data from Kenya using computer-assisted qualitative data analysis software (CAQDAS). The transcripts of interviews and focus group discussions were imported into QSR NVivo software (version 9.2) and analysed using a framework approach [35]. Layers of coding were not shared between researchers until the analysis was complete. No major inconsistencies were found between the manual and computer-assisted analyses. This allowed the Kenya analysis to serve as a benchmarking tool for the analysis of material gathered in Nigeria and Niger. The second qualitative researcher reviewed the final reports, but not the transcripts of material from Nigeria or Niger. Triangulating results using separate researchers and techniques ensures the rigor of the analytical process, enhances the credibility of the final results and is regarded as best practice.

JB and AS prepared the cross-country analyses of results and implications. Due to the considerable amount of data that was generated across the three countries, this manuscript focuses on caregiver perceptions and does not incorporate all the findings from other interlocutors (health workers and fathers). Detailed analysis of the whole data set has been synthesised elsewhere and is available as open-source UNICEF working papers [36], [37], [38].

### Ethics

Approval to conduct the research was received from the Director of the Ministry of Public Health and Sanitation in Kenya, the Ministry of Health in Niger and the National Health Research Ethics Committee of Nigeria.

Interviews were conducted at the primary carer's home and were held in as much privacy as possible. The focus group discussions were held in community areas. At the start of each interview and focus group, it was made clear to the interviewee or participants that their involvement was optional and voluntary and would not affect any future referral or medical service required or received. It was emphasised that the topic under discussion was sensitive and that all participants were free to stop the interview or leave the FGD at any point and without consequence. These measures were important in order to minimise the potential of distress or other adverse effects for the participants. The study's consent form was read and explained in detail. Informed consent was given by the signature or thumbprint of all those participating.

Data have been deposited into a secure internal database at UNICEF New York.

## Results

**Figure 4:**
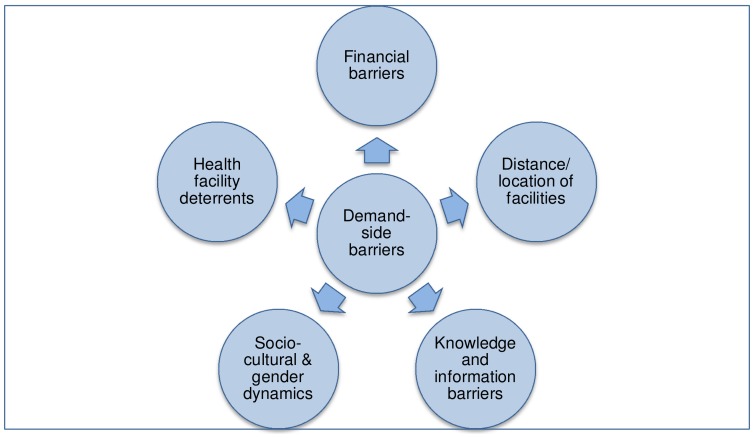
Demand-side barriers identified by participants in all three settings.

Five overarching themes were identified around which barriers to care-seeking and treatment (and related solutions) are organised in this paper. These are 1) financial barriers; 2) distance/location of health facilities; 3) socio-cultural barriers and gender dynamics; 4) knowledge and information barriers; and 5) health facility deterrents (figure 4). The relative importance of each type of barrier in various settings is described below.

### Financial barriers

Policies for free treatment services and/or medicines for children under 5 exist in all three countries, however, most participants still reported financial constraints to be a major barrier. In Kenya for example, it is national policy that treatment for children under 5 years is free at the point of delivery in government health facilities. No participant in our study was aware of this, and all thought that treatment incurred other costs (e.g. registration fees, lab tests, prescription medicine and purchasing equipment such as needles).

The main challenge is lack of money. Maybe with a disease, if you had money, then the child would be treated and they would be fine, but without money you just sit and hope the child will get better. There is nowhere you can go without money. Sometimes, even if you find money and go [to the health facility] you feel like you are wasting money because it doesn't always work, so you look around for others, those women [*nyamrerwa*] to give you other treatment. I might want to take the child to the health centre, but the doctor will want money and I don't have it. So there is nothing else I can do to help the baby. I just sit and wait for the child to get better, it can take a week for them to improve.

20 year old mother with 1 surviving child (of 3 children), Marindi, Homa Bay

Some participants in Kenya reported that they undertook direct transactions to generate money for treatment (such as selling livestock or firewood), and others borrowed money from relatives or neighbours, although this was often seen as a last resort. Several carers, however, imbued local treatments with social and spiritual value and expenditure was not always economically relative. For example, one mother asserted that whilst she did not have the means to pay 300 Shillings at the health facility, she was prepared to pay a similar amount to the *jaote* (local spiritual healer). The social and spiritual value of this expenditure ensured that, to her, it was of greater worth than the transaction to buy drugs, a material commodity, from the health facility.

In Nigeria, whilst some participants were aware that free medicine for children and pregnant women should be available at health facilities, many explained that there was rarely any stock so carers had to purchase prescribed drugs from a chemist, and also pay equipment costs and fees for lab tests. In Cross River State, one mother explained that her child had become sick whilst her husband was away, and although the mother and grandmother had tried Hausa medicine, they had been unable to find money for treatment at the health centre.

The boy was sick, his stomach came out [swollen] and he couldn't breathe. I went to the health centre, but they wouldn't help because I didn't have money. I went five times. After two weeks I found some money and brought malaria drugs and blood tonic syrup from the chemist. I was so worried about the child I wasn't thinking. Then my husband was able to borrow money from a friend because we wanted to take the child to the general hospital. We were actually on the river road [taking a boat up river to the hospital] when the child died on the river. It was the year before last, he was nine months old.

25 year old mother with 3 surviving children (of 4 children), Abi LGA, Cross River State

In both Kenya and Nigeria, participants discussed a reluctance to borrow money from their relatives or neighbours to fund treatment and many carers emphasised their inability to save money. One mother in Cross River asked, ‘*we think of saving money, but when only 200 or 300 Naira enters the house, how do you find the spare?*’ Community collection schemes reportedly existed in Cross River (some organised by women community leaders), which collected fees from families and enabled them to borrow money when essentials were needed (such as shoes for children) or during an emergency.

Only in Nigeria, were indirect costs, such as transport or losing a day's work due to attending the health centre, not raised by carers as a problematic issue.

In Niger, despite the national policy of free drugs for children under 5 and pregnant women, participants reported that there were frequent stock-outs and carers had to buy medicine on prescription. For carers who lived in a village without a health post but had travelled to a health centre and were given a prescription, further financial outlay was often required to cover additional costs of transport and purchasing the medicine at a pharmacy. Several carers reported the need to have ‘*money in hand*’ to attend a health post, and suggested that they rarely considered presenting the child if they had no money. Although some mothers received gifts of money or cash distributions, for many there was no possibility of borrowing money as they had no means of repaying the credit. As one mother explained, ‘*I am at the point that there is no money, I can't borrow, so I just sit and look up to Allah*’.

### Distance/location of health facilities

In Kenya, many participants reported that the distance to a health facility was a challenge. For these carers, access was usually by foot across difficult terrain, made more problematic during the wet season. Although some routes were navigable by bicycle or motorbike, to hire transport required additional expenditure. Therefore most carers reported that they walked, carrying their sick child.

In contrast, carers in Nigeria rarely discussed limited geographic access as a barrier to care-seeking, and only raised problems of distance and transport in relation to emergency cases or if they needed to attend a larger health facility. Similarly, in Niger, carers who lived in villages with a health post did not cite distance or location as a barrier to care-seeking, but those living in villages without a health post frequently emphasised difficulties created by the distance to and location of facilities.

You walk to the health post. If the child is small, you put them on your back and walk. It is quite far and you have to leave early in the morning. They [the health workers] are already working when you get there. If you go on the bike you get there faster, but if you don't have money, you have to walk. It doesn't depend on how serious the sickness is, it depends on the money.

Mother (age not known) with 5 surviving children (of 6 children), Bargaja, Maradi

Several participants in Niger discussed children dying during lengthy journeys to a health facility, although such accounts were generally framed in terms of poor judgement leading to delayed presentation.

### Socio-cultural barriers and gender dynamics

In spite of the fact that few socio-cultural barriers were identified with care-seeking for childhood illness in Kenya, there was an awareness of gender-related barriers, and mothers perceived the need to empower women to enable them to seek healthcare (both economically and socially). Religious beliefs were influential, not only in the prohibition of herbal medicine, but some denominations reportedly prevented treatment-seeking at health facilities. Many carers confirmed that they prayed to God or sought the services of a spiritual healer, but that this was often done in conjunction with other treatment options.

Socio-cultural barriers and gender dynamics appeared to play a larger role in care-seeking for children in Nigeria. Restrictions on the movement and societal interactions of women in Kebbi in particular, resulted in the juxtaposition of mothers as the primary caregivers and fathers as the primary care-seekers. This was a barrier to accessing effective, efficient and timely healthcare. In Cross River, fathers had little involvement with child rearing, and this, according to several participants, led to decreased responsibility and curtailed support of the family. There did not appear to be any gender-bias in treatment-seeking for children, nor was any distinction made in treating children of different wives in a polygamous marriage.

Religious barriers were also identified in both states in Nigeria. In Cross River, a number of Pentecostal denominations reportedly prohibited biomedical treatment. In Kebbi, several participants said that Imams were a source of positive health education, although there were also reports that religious authorities preached against health services. Further, rumours circulated about polio campaigns (particularly that vaccination results in infertility) remain in the community's collective memory and contribute to a negative undercurrent regarding the implementation of immunisation and other health initiatives.

In Niger, the restricted movement of women was not regarded by participants as a major barrier to care-seeking as male household heads usually gave permission for mothers and children to attend health posts. Both mothers and health workers commented, however, that fathers sometimes displayed little responsibility for their child's health because, socially and culturally, the mother was the primary caregiver. Further, while a few mothers claimed to be able to use their own money (generated through small businesses) to seek treatment, the majority were dependent on their husband's provision.

I don't know what the cost of medicine is, as the father pays. He does not tell me, only he says if he does not have the money. For the children under 5, you don't need to find money first, you can just go and then, if they write a note [prescription], you come back to find the money. Last week, I told my husband about the note and he found the money. I don't know where from, he just came back with the medicine.

Mother aged 35 with 6 children, Bokotchilli, Tillabéri

### Knowledge and information barriers

In Kenya, although information about pneumonia was lacking, participants' knowledge about causation, symptoms, prevention strategies and the availability of treatment for malaria and diarrhoea was widespread. Still, many carers reported that they wished they had access to more information in their community about childhood illnesses, home management, when to seek treatment and how much the treatment would cost. Further, participants frequently suggested that male members and ‘big people’ (the household elders) needed information too.

Some fathers do know these things, but they need to know more. They should be taught. It is important. Usually it is the women who struggle with the children, but sometimes the mothers might not be around. The fathers should be educated so that if they have children they should not say ‘I don't have money’ if the child is suffering.

Mother, aged 32 with 4 children, Lambwe, Homa Bay

In both Kebbi and Cross River in Nigeria, there was limited knowledge about the causation and prevention of childhood illnesses. Pneumonia was the least known of the three conditions, but there was also little association made between diarrhoea and water hygiene and sanitation, and similarly between malaria, mosquitoes and the use of bed nets. A lack of ability to identify risky symptoms, poor knowledge about when to seek treatment, and a perpetuating attitude of ‘wait and see’, all contributed to the late presentation of cases. In addition, the majority of carers in Nigeria called for more education on child health for themselves and also for the male members of their households and community. This was a dominant issue in Kebbi, where a mother's agency to act independently was tightly restricted and contributed to a lack of knowledge about community activities.

Some husbands can be taught, but it doesn't matter, because even if they are taught the advantages of healthcare, then they are still in the position to let the mother go or not, some may allow it, others may not, it depends on the man. They know it's a good thing to get treatment, but it is just up to the man himself.

Mother (age unknown), pregnant and with 5 other children, Koko, Kebbi

In Niger, knowledge and information about specific conditions was reasonable in both districts, although the relationship between diarrhoea, hygiene and sanitation was weak, and, as was true in the other countries, participants knew less about pneumonia than the other conditions. A number of interviewees suggested that they gained their knowledge from Allah or instinctively as a mother. Whilst the health education activities of *relais* (community health workers) was cited in many communities, several mothers commented that their meetings did not provide conducive environments for learning: many women were unable to attend; it could be difficult to hear the speaker over the noise of the collected women; and some women were too shy or intimidated to participate. In villages where there were no *relais*, the difference in the level of knowledge expressed by the participants was noticeably lower.

As was true in Kenya and Nigeria, the majority of carers in Niger stressed their need for further information and health education and lamented the lack of health education targeted at men.

### Health facility deterrents

Certain aspects of health services were identified as barriers to care-seeking in all three countries. In Kenya, important deterrents included prolonged waiting times; poor communication between staff and patients; negative previous experiences resulting in carers losing trust in health services; and a fear of being tested for HIV when presenting their child for treatment. In addition, carers were perplexed and frustrated by the lack of a reliable drug stock at their local health facility. The majority of participants volunteered examples of when they had presented for treatment only to be turned away or sent to a private chemist to purchase medication. As one mother reported,

What we are praying for, what we want, is for the government to bring the drugs to the health facilities. Life is very difficult for us because of this. You go to the health centre and they send you to the chemist, there you have to buy everything, even the needle to take back to the health centre.

Mother, aged 33 with 3 children, Ndiru, Homa Bay

In Nigeria, carers reported that the widespread polio campaign impinged on their tolerance for interaction with health services. Kebbi carers indicated that parents were ‘*at saturation point*’ with polio-related services. Distrust in biomedicine (particularly free medicine) also appeared to be widespread in Kebbi. One mother described her husband's attitude,

He does not understand why some medicine is free. Is that medicine a different standard [substandard]? Why do we have to pay for medicine for adults, and then they give medicine for the children for free? Because of poverty we don't get the vaccinations at the health centre, because when you go it costs so much money. But the other vaccines that are free [polio], he does not permit the children to have them. We don't trust anything that is free. You have to pay at the health centre, so why is it free when they come to your home? My husband doesn't have a problem going to the chemist when the child is sick, only it is the free vaccinations that he does not trust.

Mother (aged unknown) with four children, Takalau, Kebbi

Such distrust of medicine was less evident in Cross River, but there, the unclean environment of health facilities, negative attitudes of health staff, and lack of available medicines were seen by many to discourage attendance. Participants also reported that stock-outs actively encouraged carers to seek treatment from other sources. There was a sense amongst mothers in Cross River that, largely because of these supply-side issues, health centres were a place to go for immunisation and ante-natal care (ANC), but not when their child was ill.

In Niger, the dominant health facility deterrent was the lack of free medicine at the health posts. A mother in Madarounfa explained, ‘*the health post works very well for us, thanks be to Allah, the only problem is the shortage of medicine*.’ In addition, several mothers commented that the health posts were understaffed, offered limited services and that opening times restricted access, particularly at night. Interestingly, negative staff attitudes and long waiting times were not reported as deterrents to care-seeking in Niger.

### Solutions suggested by participants

A key focus of the research was to elicit participants' own solutions to the barriers they identified in order to promote those that were both relevant and acceptable to local caregivers. Tables 3–5 present a summary of the identified barriers and corresponding solutions from each of the three countries.

**Table 3:** Barriers and related solutions identified by caregivers in Kenya.

**Financial**	Identified barriers:	Lack of money
		Inability to save
		Cost of treatment (direct and indirect)
		Limited empowerment of women (economically)
	Suggested solutions:	Ensure that the national policy to provide free treatment at the point of delivery for children under 5 is put into effect in all government health facilities
		Publicise fixed costs at health facilities to help families budget the necessary funds and to deter health workers from overcharging or incorporating false costs
		Support initiatives to make women more financially independent and enable them to seek treatment without relying on their husbands, family or having to look for money first themselves
		Develop community funds into which villages or groups of families can pool resources to provide each other with financial support when a child is ill
**Distance and location**	Identified barriers:	Long distances to facilities
		Location of facilities in difficult-to-reach areas
		Lack of transport
	Suggested solutions:	The community should better maintain paths and walkways, and village chiefs and clan elders should encourage constituents to improve road access using the Constituency Development Fund (CDF)
		Health facilities should put transport options (such as a motorbike ambulance) in place to assist patients to travel from home to the health centre, particularly in emergency situations
		Health facilities should implement mobile clinics and better outreach activities to provide services to remote communities and villages
**Socio-cultural and gender dynamics**	Identified barriers:	Insufficient opportunities for health education
		Preference for home management and use of local herbal treatments
		Poor school environment
		Dependency on spiritual healers
		Religious prohibition of treatment-seeking
		Limited empowerment of women (socially)
	Suggested solutions:	Introduce more community education and sensitisation (including spiritual leaders and local healers) to counter religious beliefs that preclude treatment-seeking and to encourage people not to use local herbal treatments
		Socially empower women and educate men and community leaders to facilitate women's attendance at health facilities
**Knowledge and information**	Identified barriers:	Lack of strategic and targeted health education
		Poor communication strategies
	Suggested solutions:	Provide more frequent health education opportunities in the community (in group settings and within households)
		Community health workers can be ‘positive change agents’ and to act as links between the community and the local health facility
		Communication strategies should be developed in collaboration with local stakeholders so that key health messages are relevant, appropriate and delivered in an engaging way
**Health facility deterrents**	Identified barriers:	Waiting times
		Poor communication and negative staff attitudes
		Other issues (HIV testing, clinic card)
		Supply-side issues (lack of drugs, equipment and diagnostic capability; limited follow-up and tracing; curtailed activities of CHWs)
	Suggested solutions:	Identify ways to improve the attitude and behaviour of health staff towards patients and reduce waiting times and support better patient flow
		Guarantee a reliable supply of drugs to health facilities

**Table 4:** Barriers and related solutions identified by caregivers in Nigeria.

**Financial**	Identified barriers:	Lack of money
		Cost of treatment (direct and indirect)
		Inability to save (distrust of others)
		Limited empowerment of women (economically)
	Suggested solutions:	Publicise fixed treatment costs in order to deter chemists from offering price flexibility dependent on brand, quality and dosage of drugs purchased, and to deter health staff from over-charging or incorporating false costs
		Develop a community-based scheme to support families and children, where pooled resources are shared amongst members when needed (Kebbi)
		Develop a Community Health Insurance Scheme (Cross River)
		Support initiatives to economically empower women
**Distance and location**	Identified barriers:	Long distances to facilities
		Location of facilities in difficult-to-reach areas
		Lack of transport
	Suggested solutions:	Increase door-to-door service delivery as his is a system that has gained traction with the community as a result of the polio campaign (Kebbi)
		Health facilities should provide ambulance services, especially for emergencies
**Socio-cultural and gender dynamics**	Identified barriers:	Limited knowledge on causation, prevention and treatment for childhood illness
		Use of local treatments (especially for pneumonia)
		Religious prohibition of treatment-seeking
		Dependency on spiritual healers (CR)
		Limited empowerment of women (socially)
	Suggested solutions:	Educate and sensitise religious leaders (both Christian and Islamic) and spiritual healers to promote health facility attendance and (in Kebbi) to overcome negative rumours about biomedical treatment
		Work within local leadership structures to overcome socio-cultural barriers, particularly if influential community members led by example
		Convey important child health messages through churches and pastors (Cross River) and mosques and imams (Kebbi)
		Engage with all sectors of the health system (including local healers, traditional birth attendants and patent medicine vendors) to advocate at the community level and prioritise health facility attendance above the use of local treatment
		Socially empower women in parallel to the education of men (both states)
**Knowledge and information**	Identified barriers:	Divergence between caregiver/care-seeker roles (K)
		Lack of strategic and targeted health education at community level
	Suggested solutions:	Provide more strategic, targeted and sustained health education (both states): educate all members of the community (not just parents), have a women educator teach women at home (particularly in Kebbi as a way to overcome restrictions on movement), and gather men for community meetings on child health
**Health facility deterrents**	Identified barriers:	Conflicting messages about free treatment
		Distrust of biomedicine
		Health centre environment and attitude of staff
		Perception health facility for immunisation, ANC but not treatment of childhood illness (CR)
		Supply-side issues (lack of drugs, equipment and diagnostic capability, limited tracing and follow-up, lack of government support for primary healthcare)
	Suggested solutions:	Improve the environment of health facilities and the attitude of staff (so that they are sensitised and do not quarrel with their patients)
		Resolve conflicting messages about the availability of free medicine and publicise fixed costs
		Ensure a reliable supply of free medicine to be available at the health facility

**Table 5:** Barriers and related solutions identified by caregivers in Niger.

**Financial**	Identified barriers:	Lack of money
		Cost of medicine on prescription (direct and indirect)
		Limited empowerment of women (economically)
	Suggested solutions:	Economically empower women (small-scale businesses for women were raised as a solution to financial constraints more often in Madarounfa than in Kollo)
**Distance and location**	Identified barriers:	Distance (in villages with no health post)
		Location (far from pharmacy, particularly for villages with no health post)
		Lack of transport
		Limited outreach
	Suggested solutions:	Establish health posts in villages that currently do not have one and ensure they have a reliable supply of free medicine to overcome challenges of access associated with onwards referral to a pharmacy
		Expand routine outreach services and improve the support for health workers to provide these services
		Expand the role of *relais* so that they continue to provide health education and support, but also carry out tracing and follow up, and actively encourage uptake of health services
**Socio-cultural and gender dynamics**	Identified barriers:	Lack of support and responsibility from some male household heads in care-giving and care-seeking
		Use of traditional practices including plant medicine and spiritual healers
		Limited empowerment of women (socially)
	Suggested solutions:	Parallel to the social and financial empowerment of women, encourage men to take responsibility and provide adequately for their wives and children
		Promote greater involvement of the village chief and local leadership structures to ‘lead by example’
		Encourage health facility attendance whilst educating the community not to be reliant on local healing practices such as plant medicine or traditional and spiritual healers
**Knowledge and information**	Identified barriers:	Limited knowledge of causation, prevention and treatment (diarrhoea and pneumonia)
		Lack of strategic and targeted health education at community level
		No *relais* in villages with no health post
	Suggested solutions:	Recruit and train more male *relais* to encourage engagement with fathers and household heads, and to provide a positive role model for male members of the community
		Conduct regular health education activities targeted at fathers and household heads (separately from women)
		Conduct regular health education activities for women in their homes
		Support the work of *relais* who are important sources of information and are very helpful, both in terms of delivering key messages, and supporting care giving and care-seeking
**Health facility deterrents**	Identified barriers:	Perception that health post is understaffed
		Restricted opening times, particularly at night
		No admission and limited services
		Supply-side issues (lack of drugs, equipment and diagnostic capability; limited tracing and follow-up)
	Suggested solutions:	Ensure a reliable supply of drugs at the health post, particularly free medicine for children under 5
		Support community investment in the health post
		Introduce incentives (such as plumpy nut distribution) to encourage attendance
		Provide adequate resources to health workers and *relais* (accommodation and transport for outreach)

Suggested solutions to the various barriers included community-level, facility-level and more policy-oriented actions, as well as actions to change underlying issues such as social perceptions and gender dynamics. Selected solutions are highlighted below.

In relation to financial barriers, solutions suggested in Kenya included: the organisation of community funds that could be accessed by vulnerable families during emergencies; maintaining a stable supply of free medicines and transparency about fixed costs at facilities; ensuring that the national policy for free health care is put into effect; and economically empowering women to make them more financially independent. In Niger, many carers reported that engagement with the monetary economy was a continuous struggle and the possibility of saving money or contributing to a community-funding scheme to access healthcare was impossible. Only a few mothers discussed cash distributions, and none suggested this as a way of overcoming their financial constraints. There were concerns that any money earned would immediately be absorbed into general household funds, but several mothers who were already engaged in small-scale business discussed having their own money and being free to spend it on essential items, including a child's healthcare, as they saw appropriate. Other respondents suggested that even if a mother's money was absorbed, the level of household income would rise, and therefore financial barriers may ease.

Regarding distance and location barriers, carers in Kebbi in Nigeria suggested that a door-to-door service would allow them to better engage with healthcare for their children as it would increase their ability to directly access services despite the restrictions on their movement outside the household compound.

A common solution offered in all three countries to resolve barriers relating to socio-cultural issues and gender dynamics was to socially empower women. In Niger, this was suggested in parallel with health education for men so that they would be ‘sensitised’ to the health needs of their family, and would permit and support their wives to attend the health post when necessary.

Solutions to knowledge and information barriers focused on ensuring that health education efforts were sustained, regular and more strategic in terms of targeting key members of the community who had decision-making power and influence (especially men and local leaders). In Niger, many participants cited the important work of *relais*, and those from villages without active *relais* all confirmed that having *relais*, with or without a heath post in the vicinity, would be of huge benefit to the community.

Finally, the suggested solutions to health facility deterrents focused on developing measures to improve patient experiences, specifically to reduce waiting times, improve interactions with health staff, and ensure that drugs were in stock.

## Limitations

This study is subject to a number of limitations. Risks associated with misinterpretation are inherent in consecutive translation, but a number of strategies were used to improve accuracy. In translating between English and the various local languages, we planned translation and interpretation styles in advance and decided how to best capture colloquialisms, abstractions, idiomatic expressions and jargon. We used short units of speech and careful phraseology that were refined during the finalisation of the interview and focus FGD frameworks. The research team validated sections of narrative that were transcribed *ad verbatim* and certain responses were reiterated to participants for clarification and confirmation. Full transcriptions of all interviews and FGDs were made by the research assistants and included the translation and back-translation of both questions and responses. During the first phase of analysis, transcripts were cross-referenced with the research team's notes and any areas of digression highlighted and discussed. The research team had full visibility of the growing data and were able to query potential anomalies throughout the study. This served to mitigate the risk of errors in the translation and transcription process.

Second, it is possible that interviewees expressed what they perceived to be appropriate or socially desirable responses. This is a risk in qualitative research generally, but was not seen to be a major limitation in this study because we conducted informal, private interviews; the interviewees did not know the research team; and the semi-structured interview format allowed questions to be asked in multiple ways and responses triangulated. The FGDs also provided data sets similar to those in the individual interviewees and this strengthened their validity.

It is important to note that because participants were selected by local community health workers or identified through their collaboration with the research team (as was the case for most of the health professionals who participated in FGDs), there was the potential for selection bias. There may be some predisposing factors about these individuals as opposed to individuals who were not previously known to community health workers or the research team. Although relatively small, however, the sample size resulted in saturation of findings. This lessened the impact of purposive non-probability sampling. The results are likely representative of the population in the districts were the research was located, but are not necessarily generalisable to the various regions, and cannot be extrapolated to wider country contexts.

## Discussion

Although previous studies appertaining to the study sites were scarce (only one for Cross River State, Nigeria [17] and one in Maradi Region, Niger [24]), many of the barriers identified through this research are similar to those identified across other low-resource settings in sub-Saharan Africa [3]–[6], [13]–[15], [18]–[22], [25]. These include financial barriers; distance to facilities; lack of knowledge about available services; and perceived shortcomings of facilities, particularly frequent drug stockouts.

Still, there are important distinctions to be made between the findings of this research and previous studies, and across the three sites. In this research, religious beliefs (such as the prohibition of biomedical treatments) were revealed as barriers in Kenya and Nigeria, but not Niger, and had not been identified by previous studies on care-seeking from these three countries. A lack of knowledge about illness severity was identified among caregivers in only one previous study in Kenya [15], yet in this research, it was found only to be a barrier in Niger. Further, whilst social norms around household decision-making and gender relations had been previously identified in Cross River, Nigeria, this research revealed that it was also a problem in Kebbi State, Nigeria where women's movements and social interactions were severely restricted, and in Niger where fathers had decision-making authority but little involvement in care-giving.

The presentation of locally relevant, acceptable and appropriate ‘solutions’ generated by caregivers in these settings is also new to the literature, and again there are nuanced differences across the sites. For example, in response to the problem of negative interactions between health workers and families, caregivers in Kenya suggested improving the attitude and behaviour of staff towards patients, whereas in Niger, suggestions focused on community investment in the health post and providing adequate resources (including accommodation and transport) to health workers and the community-based *relais*. Participants in Niger perceived that these measures would have a significant impact on patient attendance, compliance and positive engagement with health services. Although lack of knowledge of danger signs was seen to be a problem among men in all three countries, only in Niger did participants suggest that male community-based workers (*relais*) should be purposefully recruited as a way to engage men in issues relating to child health.

The majority of the solutions suggested are not likely to be prohibitively expensive for Ministries of Health and their local counterparts to implement. Many of the solutions focused on engaging the community as active partners (e.g., developing community emergency funds, being transparent about fixed treatment costs available at public facilities and local pharmacies, promoting community maintenance of paths and walkways, and engaging local leaders and informal providers to improve their knowledge). Other suggestions focused on expanding community-based services (through mobile services, better outreach, or enhanced roles for community health workers) and such interventions have proved to be cost-effective in other settings [39], [40], [41]. Further, the governments of all three countries have indicated that community participation and promotion of community-based services are national priorities, so such solutions are likely to be politically appealing as well.

Solutions that may be more difficult to implement are those designed to address health facility deterrents such as long wait times, poor interactions between staff and patients and supply side issues including drug stock outs. These problems have been found to be pervasive in other settings that have also abolished user fees [42], [43]. Solutions intended to address socio-cultural and gender barriers such as economically and socially empowering women may also be problematic for the health sector to achieve. However, the landmark ‘Intervention with Microfinance for AIDS and Gender Equity’ (IMAGE) study in South Africa (which resulted in a dramatic reduction in intimate partner violence after just two years) offers a precedent for linking social and economic development interventions with improved health outcomes [44].

Demand-related barriers to child health interventions have often been neglected or insufficiently considered in research, policy and programming. Many research questions remain, including how to better use existing data sources to capture local demand for services for childhood illnesses and the associated barriers, as well as what innovations (including multi-sectoral innovations) might be used to help address socio-cultural and gender barriers in settings where women bear responsibility for rearing children, without independent authority to obtain the care their children may need.

The findings of this study suggest that barriers to care-seeking for childhood illness and the related solutions are unique to specific contexts and may resonate with intended beneficiaries in different ways. As such, understanding what the local drivers are is central to the development of effective and sustainable interventions that are rooted in the lived experience of the intended beneficiaries. Further, the types of barriers and solutions identified in this study suggest that efforts to generate demand must be synergised with efforts to improve the supply of child health services. Families who expend time, energy and resources to seek care for childhood illnesses should not be denied care because of stock-outs, staff shortages or limited hours of operation.

Each of the settings studied in the three countries are diverse and face particular challenges to health and healthcare. Significant advancements are possible, however, when communities participate in both problem identification and resolution, and are actively engaged as important partners in improving child health and survival.

## Supporting Information

Information S1 **Semi structured interview framework – carers of children under 5.**
(DOCX)

Information S2 **Focus group discussion framework – community health workers.**
(DOCX)
