# Supplementary material for: Local Barriers and Solutions to Improve Care-Seeking for Childhood Pneumonia, Diarrhoea and Malaria in Kenya, Nigeria and Niger: A Qualitative Study
Source: PLoS One. 2014 Jun 27;9(6):e100038. doi: 10.1371/journal.pone.0100038 (PMC4074042; doi:10.1371/journal.pone.0100038)
Supplement: Information S1 — Semi structured interview framework – carers of children under 5. (DOCX) [file pone.0100038.s001.docx]

**Information S1: Semi structured interview framework – carers of children under 5**

**Demographic details**

- Age
- Relationship to child
- Marital status
- Number of children in care
- Age of children
- Gender of children
- Do children go to school
- Did carer go to school
- Does family (paternal, maternal) live near
- Religion
- Employment
- General income range

**Q1**

What are the main child health problems in your community?

Do many young children (under 5) die in this area?

What do they die from?

**Q2**

What do you call malaria in your community?

What causes malaria and what are the symptoms?

How do you prevent malaria and do you do this?

Do your family sleep under bed nets?

Where did you get your family’s bed net(s) from? Were they treated? How much did they cost?

Do bed nets have any other uses?

If the children sleep under a bed net, when and how do they get malaria? What can be done?

What do you call diarrhoea in your community?

What causes diarrhoea and what are the symptoms?

How do you prevent diarrhoea and do you do this?

Where does your family get water from? Do you drink it directly from the source?

Where does your family (adults and children) go to the toilet? (If in the bush, do you leave it?)

Does your family (adults and children) wash their hands? With water only?

What do you call pneumonia in your community?

What causes pneumonia what are the symptoms?

How do you prevent pneumonia and do you do this?

**Q3**

For child illness, do you use traditional medicine / home remedies?

What for? How do you prepare? Where do you get the herbs? Who should you how to use them?

Does your family use a traditional doctor / spiritual healer for child illness?

What for? Do you have to pay? How much?

Do you sometimes go to the health centre for child illness?

How far is the health centre from your place? How do you get there?

What cost is incurred to visit the health centre and obtain medicine?

What is your opinion about the quality of the services provided by the health centre?

Do you have to wait to be seen at the health centre?

Do you sometimes use the chemist for child illness?

Why do you use the chemist (instead of the health centre?)

How far is the chemist from your place? How do you get there?

What cost is incurred to visit the chemist and obtain medicine?

**Q4**

When a child is ill, who do you tell?

What kind of help does your husband / family provide to you when a child is ill?

Who takes the decision to treat the child?

**Q5**

What cultural beliefs influence child illness and treatment seeking in your community?

**Q6**

How often is your child ill?

How often do you get treatment for your child?

When did you last visit the health centre because of child illness? (Elicit narrative)

**Q7**

Of the three illnesses, which is the most dangerous / serious for children in your opinion?

Of the three illnesses, which are you most likely to visit a health centre for?

**Q8**

Where do you get your information about child illness?

What child survival information, education and communication activities are targeted at mothers?

What measures should be taken to improve the community’s knowledge about child illness?

**Q9**

What are the main challenges your family faces in going to the health centre or accessing treatment for child illness?

What are the reasons that some families not take their child for treatment if they are ill?

Does the cost of accessing treatment sometimes prevent you taking the child?

**Q10**

What are the solutions to these challenges / barriers?

**Q11**

What can be done to improve the health of children in this area?
