# Supplementary material for: Local Barriers and Solutions to Improve Care-Seeking for Childhood Pneumonia, Diarrhoea and Malaria in Kenya, Nigeria and Niger: A Qualitative Study
Source: PLoS One. 2014 Jun 27;9(6):e100038. doi: 10.1371/journal.pone.0100038 (PMC4074042; doi:10.1371/journal.pone.0100038)
Supplement: Information S2 — Focus group discussion framework – community health workers. (DOCX) [file pone.0100038.s002.docx]

**Information S2: focus group discussion framework – community health workers**

(NB: Similar frameworks were created for mothers of children under 5, fathers of children under 5 and other community members)

**Q1**

What are the main child health problems in your community?

Do many young children (under 5) die in this area?

What do they die from?

**Q2**

What causes malaria and what are the symptoms?

How do you prevent malaria and do you do this?

If they children sleep under a bed net, when and how do they get malaria? What can be done?

What causes diarrhoea and what are the symptoms?

How do you prevent diarrhoea and do you do this?

What causes pneumonia what are the symptoms?

How do you prevent pneumonia and do you do this?

**Q3**

For child illness, do some families use traditional medicine / home remedies? What for?

Do some families use a traditional doctor / spiritual healer for child illness?

Do some families go to the health centre for child illness?

What is your opinion about the quality of the services provided by the health centre?

Do some families use the chemist for your child illness?

Why do they use the chemist (instead of the health centre?)

**Q4**

What kind of help do husbands / family provide to mothers when a child is ill?

**Q5**

What cultural beliefs influence child illness and treatment seeking in this community?

**Q6**

Where do the community learn about child illness?

What child survival information, education and communication activities are targeted at mothers/fathers?

What measures should be taken to improve the community’s knowledge about child illness?

**Q7**

What activities do health workers undertake in the community?

What challenges do health workers face doing their work in the community?

Do you get support from the health centre, the government, the community?

**Q8**

What are the main challenges families in this community face in going to the health centre or accessing treatment for child illness?

What are the reasons that some families not take their child for treatment if they are ill?

Does the cost of accessing treatment sometimes prevent some families from taking the child?

**Q9**

What are the solutions to these challenges / barriers?

**Q10**

What can be done to improve the health of children in this area?
